# Recipient Minus Donor Body Mass Index Difference and One-Year Graft Function After Living-Donor Kidney Transplantation

**DOI:** 10.3390/jcm15218298

**Published:** 2026-10-25

**Authors:** Eren Eynel, Idris Oruc, Hasan Ince, Enver Yuksel, Ramazan Danis

**Affiliations:** Department of Nephrology, Gazi Yaşargil Training and Research Hospital, Diyarbakır 21070, Türkiye; dridris21@hotmail.com (I.O.); drhasanince@gmail.com (H.I.); ferhat_enver@hotmail.com (E.Y.); dr.r.danis@gmail.com (R.D.)

**Keywords:** kidney transplantation, living donor, body mass index, anthropometry, estimated glomerular filtration rate, graft outcomes

## Abstract

**Background:** Recipient minus donor body mass index (BMI) difference is an anthropometric exposure whose anatomical validity is unproven. We evaluated its association with 1-year graft function after living-donor kidney transplantation. **Methods:** This retrospective cohort included all 249 adult living-donor kidney transplant recipients at one center during 2013–2024. Adjusted linear regression assessed 1-year estimated glomerular filtration rate (eGFR) among recipients alive with functioning grafts. The sensitivity, 6-month, longitudinal, ranked-composite, and clinical-event analyses were exploratory, without adjustment for multiple comparisons. **Results:** All 14 recipients without 1-year eGFR had experienced graft loss or death, leaving 235 for the primary analysis. The adjusted estimate was −0.55 mL/min/1.73 m^2^ per kg/m^2^ BMI difference (95% confidence interval [CI] −1.06 to −0.05; *p* = 0.033), equivalent to −2.77 (95% CI −5.31 to −0.23) per 5 kg/m^2^. Alternative BMI parameterizations showed similar fit. Exploratory eGFR estimates varied by visit and model specification. The unadjusted graft-loss/death hazard ratio was 0.88 per 5 kg/m^2^ (95% CI 0.55–1.42; 14 events). The full-cohort ranked-composite interval included zero. **Conclusions:** The adjusted eGFR association describes survivors with functioning grafts; complementary exploratory analyses do not change this conditional interpretation. Residual confounding remains possible. BMI difference does not quantify anatomical size mismatch, and these findings do not establish causality or donor-selection thresholds.

## 1. Introduction

Kidney transplantation is the preferred treatment for eligible patients with end-stage kidney disease, providing better survival and quality of life than maintenance dialysis [1,2]. Nevertheless, post-transplant graft function varies considerably, and identifying donor–recipient characteristics associated with this variation remains clinically important [3].

Beyond immunological compatibility, donor and recipient age, sex, body size, and metabolic characteristics may influence allograft performance [4,5,6]. Anatomical donor–recipient size mismatch concerns the relationship between the transplanted organ and the recipient’s requirements. An arithmetic difference in BMI is a distinct anthropometric exposure whose anatomical validity cannot be assumed.

The nephron underdosing hypothesis proposes that transplantation of a relatively small kidney into a recipient with higher metabolic requirements may lead to adaptive hyperfiltration, glomerular hypertension, and progressive allograft injury [7,8,9]. Consistent with this hypothesis, donor–recipient weight ratio, body surface area mismatch, kidney size, and sex mismatch have been associated with graft outcomes in several cohorts [10,11,12].

Body mass index (BMI) standardizes weight by squared height, whereas donor–recipient weight or body surface area (BSA) ratios compare absolute body size. These anthropometric measures remain indirect estimates of the balance between nephron supply and recipient demand [10,11,12]. Computed tomography (CT)-derived kidney volume provides anatomical information closer to the transplanted organ itself, although volume does not quantify the functioning nephron number [13]. BMI cannot distinguish lean mass from adiposity, and identical BMIs may occur in people with different heights and absolute body sizes. Recipient or donor obesity may also affect outcomes through distinct pathways [14,15,16,17,18,19].

We selected recipient minus donor BMI difference as an available anthropometric exposure because both BMI values were routinely recorded. Source height, weight, body-composition measurements, and kidney imaging or weight measurements were unavailable in the analytic dataset. Subtraction retains direction and expresses the association per kg/m^2^ without imposing an arbitrary threshold. The BMI ratio instead represents proportional differences relative to donor BMI. Neither choice reconstructs absolute weight, BSA ratios, or anatomical kidney size. The original difference specification was retained for the primary analysis, while the less restrictive model containing recipient and donor BMI separately was examined explicitly; the difference was not assumed to be biologically superior.

Living-donor transplantation offers a comparatively homogeneous setting for examining non-immunological donor–recipient characteristics [20,21]. Previous deceased-donor research has evaluated BMI difference without identifying increased graft-failure risk [22]. The present study adds an outcome- and setting-specific evaluation of early graft function in living-donor pairs; it does not introduce a new mismatch measure or establish its anatomical validity.

We therefore evaluated whether recipient minus donor BMI difference was associated with 1-year eGFR among recipients alive with a functioning graft after living-donor kidney transplantation. The directional hypothesis was that a higher recipient BMI relative to donor BMI would be associated with lower eGFR. This anthropometric hypothesis does not establish equal and opposite biological effects of the two BMI measures or directly test anatomical nephron underdosing. Graft loss or death within the first year was examined as an exploratory secondary outcome. Sensitivity analyses assessed the exposure’s direction, functional form, component measures, and additional adjustment for donor eGFR.

## 2. Materials and Methods

### 2.1. Study Design and Participants

This retrospective cohort included all 249 adults who underwent living-donor kidney transplantation at Gazi Yaşargil Training and Research Hospital between January 2013 and December 2024. These 249 recipients constituted all living-donor kidney transplants performed at the center during the study period, and every recipient was included in the cohort. No recipient was excluded because recipient or donor BMI or required baseline covariate data were unavailable. All recipients underwent their first living-related-donor transplantation; all transplants were ABO-compatible, and no marginal living donors were included. No a priori sample-size calculation was performed. Death or graft loss during follow-up did not exclude recipients from the cohort or full-cohort analyses; it precluded subsequent graft-function assessment.

All kidneys were obtained from living related donors who had passed the institutional and legally required living-donor evaluation. All donors had normal serum creatinine concentrations and no known kidney disease. Quantitative donor creatinine and derived eGFR are summarized in Table 1.

The study is reported in accordance with the Strengthening the Reporting of Observational Studies in Epidemiology (STROBE) recommendations.

### 2.2. Immunosuppression

Induction therapy was administered selectively at the discretion of the transplant council, based on donor–recipient human leukocyte antigen (HLA) compatibility. Recipients requiring induction received antithymocyte globulin (ATG-F; Fresenius, Bad Homburg vor der Höhe, Germany), initiated before entry into the operating room and continued at 1–2 mg/kg/day. Treatment duration was determined by the treating physician according to clinical and laboratory parameters.

Oral maintenance immunosuppression consisted of tacrolimus, mycophenolate mofetil, and prednisolone. Tacrolimus was initiated at 0.15 mg/kg/day and titrated to a target blood concentration of 10 μg/L during the first month and 7–10 μg/L during the remainder of the first year. Blood tacrolimus concentrations were measured periodically to guide dose adjustment. Mycophenolate mofetil was administered at 1000 mg twice daily; the dose was reduced or the drug withheld for neutropenia (absolute neutrophil count <1.3 × 10^3^ cells/μL) and adjusted for gastrointestinal adverse effects.

Methylprednisolone 500–1000 mg was administered intravenously on day 0. The dose was subsequently reduced by half; this was followed by conversion to oral corticosteroid therapy on postoperative day 3. Oral prednisolone was tapered to 5 mg/day by the beginning of the third post-transplant month and continued as maintenance therapy. Follow-up was conducted according to institutional protocols. Rejection episodes were treated according to rejection type.

### 2.3. Data Collection and Exposure Definition

Demographic and clinical data were extracted from institutional electronic records. Available prespecified covariates were recipient age and sex, donor age and sex, pretransplant dialysis duration and status, HLA mismatch number, recipient hypertension, and recipient diabetes mellitus.

Preemptive transplantation was represented by a binary indicator. Dialysis duration was recorded in months and entered as 0 for all 95 recipients who had never received dialysis. Both terms were included to allow a linear duration association among previously dialyzed recipients and a separate preemptive-status contrast at zero dialysis duration. Thus, the preemptive coefficient is a model-based contrast at zero months, not the average difference between the two groups. The terms are not algebraically redundant, because dialysis duration varies among previously dialyzed recipients; collinearity was assessed using variance inflation factors.

Qualitative cohort characteristics included donor kidney health, panel-reactive antibody (PRA) and donor-specific antibody (DSA) status, ABO compatibility, first-transplant and living-related-donor status, absence of marginal donors, and delayed graft function (Supplementary Table S1). Donor baseline serum creatinine, age, and sex were available for all 249 donors. Donor eGFR was calculated using the 2021 race-free CKD-EPI creatinine equation [23], based on serum creatinine measured before donor nephrectomy, with creatinine in mg/dL and recorded donor age in years. Donor serum creatinine and derived eGFR were summarized descriptively and constituted the only available donor functional information. No additional donor functional measurements, including measured GFR, 24 h creatinine clearance, or split renal function, were available in the original clinical records accessible for this study. Recipient primary kidney disease, quantitative PRA/cPRA results, antibody assay details, and individual cold and warm ischemia times were unavailable for analysis. Uniform qualitative characteristics were not included as adjustment terms because they had no between-patient variation.

Donor and recipient BMI were assessed immediately before transplantation. For recipients receiving dialysis, BMI was calculated using the most recent dry weight.

Patient-level induction receipt, duration and cumulative ATG-F dose; longitudinal maintenance doses and measured tacrolimus concentrations; and major treatment changes were not captured in the analytic dataset. Exposure to alternative agents, including everolimus, could not be quantified; the protocol targets do not establish achieved drug exposure.

Post-transplant rejection occurrence, recorded clinical type, and month after transplantation were summarized descriptively. Rejection status was available for all recipients; type and timing were available for every recipient with recorded rejection. Biopsy confirmation and histological grading were not available in the analytic dataset. The primary model was restricted to baseline covariates; rejection was a post-transplant event and was reported descriptively rather than entered as a baseline adjustment term.

Recipient minus donor BMI difference was calculated as recipient BMI minus donor BMI. Positive values indicated that recipient BMI exceeded donor BMI. The exposure was analyzed continuously in the primary analysis. Its distribution and range and the proportion of pairs with a positive difference were described. The sign indicates the relative BMI values only and does not establish anatomical kidney undersizing or distinguish lean mass from adiposity. Recipient and donor BMI distributions were summarized using categories of <18.5, 18.5 to <25, 25 to <30, and ≥30 kg/m^2^ and paired plots. Source height, weight, waist circumference, body composition, kidney or cortical volume, and kidney weight were not captured. Thus, weight and BSA ratios could not be derived, and BMI difference could not be validated against anatomical size mismatch.

### 2.4. Outcomes

The primary outcome was 1-year eGFR recalculated using the 2021 race-free Chronic Kidney Disease Epidemiology Collaboration (CKD-EPI) creatinine equation [23]. The same equation was applied at discharge and 6 months. Individual measurement dates were unavailable, so visits were nominal and actual assessment windows could not be verified. Recipient age was approximated by recorded baseline age at discharge and by baseline age plus 0.5 and 1 year at the follow-up visits, respectively. Nonnumeric laboratory entries and measurements nominally occurring after recorded death or graft loss were excluded. The 1-year analysis therefore estimates an association conditional on survival with a functioning graft and an available valid measurement. All eGFR calculations were creatinine-based; cystatin C was not available and was not incorporated into the equations.

The exploratory clinical outcome was the first occurrence of graft loss, or all-cause death within 12 months after transplantation. Graft loss was defined as return to dialysis or retransplantation. Component events and their overlap were described separately. Event times were available as months after transplantation. Recipients with a valid 12-month measurement and no earlier recorded event were censored at 12 months. All 249 recipients were included in clinical event analyses. Recorded causes of first-year deaths were summarized descriptively; infection, myocardial infarction, and cerebrovascular event labels were retained without inferring diagnostic subtypes.

### 2.5. Statistical Analysis

Continuous variables are presented as mean ± standard deviation (SD) or median with interquartile range (IQR); categorical variables are reported as number and percentage. Variable-level availability is reported in Supplementary Table S1. All 249 recipients had complete recorded exposure and baseline model covariates. Cross-sectional eGFR analyses used valid available measurements without imputation. The adjusted 1-year BMI-difference model was the original primary analysis; estimates for its other covariates were exploratory. Sensitivity analyses, alternative BMI/eGFR specifications, 6-month comparisons, longitudinal models, and the ranked composite were post hoc and exploratory. Clinical-event analyses were exploratory secondary analyses. Nominal two-sided *p* values and 95% confidence intervals (CIs) are reported without adjustment for multiple comparisons. The primary coefficient and CI were also rescaled from unrounded estimates to a 5 kg/m^2^ contrast. No clinically meaningful eGFR margin was prespecified, and no post hoc power calculation was performed.

The association between BMI difference and 1-year eGFR was evaluated using univariable and multivariable ordinary least-squares linear regression. The adjusted model included recipient age, donor age, recipient sex, donor sex, dialysis duration, preemptive transplantation, HLA mismatch number, hypertension, and diabetes mellitus. Because only two recipients had undergone peritoneal dialysis, dialysis modality was reported descriptively but was not modeled as a separate multicategory covariate. For visualization, observed eGFR values and the unadjusted fitted mean were plotted against BMI difference. Adjusted mean predictions were averaged over the baseline covariate distribution of the 235 primary-analysis recipients. Both curves were accompanied by pointwise 95% confidence intervals for the estimated mean.

Model assumptions for the primary linear regression were assessed using residual diagnostics, the Breusch–Pagan test, and variance inflation factors. The original adjustment set was retained; a sensitivity model additionally included donor eGFR. The difference model is a restricted form of the model containing recipient and donor BMI separately: its single BMI coefficient assigns equal-magnitude, opposite-sign coefficients to the two component BMIs. This is a mathematical restriction, not evidence of equal and opposite biological effects. The two models used the same 235 recipients and adjustment variables. A one-degree-of-freedom partial F test assessed the restriction. Adjusted R^2^, Akaike and Bayesian information criteria (AIC and BIC), in-sample root mean squared error (RMSE), and leave-one-out cross-validated RMSE assessed fit and internal prediction error (Supplementary Table S2). Other sensitivity analyses examined positive versus nonpositive difference, quadratic and cubic terms, laboratory-reported eGFR, and 6-month estimates in all evaluable recipients versus the 1-year analysis population (Supplementary Table S3 and Table 2).

A linear mixed-effects model used all available pre-event eGFR measurements at discharge, 6 months, and 12 months, with recipient-specific random intercept, categorical visit, BMI difference, and BMI-difference-by-visit interactions, and the same baseline covariates as the primary model. Residual variances were allowed to differ by visit. The model was fitted by restricted maximum likelihood; visit-specific slopes used Wald confidence intervals, and the two interaction terms were assessed jointly. A common-residual-variance model was fitted as a sensitivity analysis. The longitudinal likelihood assumes an ignorable observation process conditional on the model; it does not resolve informative dropout or define graft function after death or graft loss [24].

A post hoc ranked-composite analysis retained all 249 recipients, using a worst-rank approach to outcomes truncated by death [25]. Recipients who had died by 12 months were ranked worst, those alive with graft loss next, and subsequently, those alive with functioning grafts were ranked by 12-month eGFR, in increasing order. Regression used the original baseline covariates, with effects expressed as percentile-rank points per 5 kg/m^2^ BMI difference and percentile 95% CIs from 5000 participant-level bootstrap resamples. The hierarchy, rank scaling, and bootstrap procedure are detailed in Supplementary Table S5. This analysis concerns a different outcome from eGFR and does not assign graft function after death or graft loss.

Kaplan–Meier estimates summarized survival free of graft loss or death, with log–log confidence intervals. Univariable Cox regression used BMI difference per 5 kg/m^2^ and Efron handling of tied event months. A BMI-difference-by-log-time interaction assessed the proportional-hazards assumption, recognizing the low power of this assessment. With only 14 composite events, an adjusted event regression was not undertaken.

For graft loss, death before graft loss was treated as a competing event, and Aalen–Johansen cumulative incidence functions were estimated. In the two recipients with both events recorded in the same month, graft loss preceded death; each therefore contributed a graft-loss event to the competing-risk analysis. There was no censoring before 12 months, so endpoint cumulative incidences equal event proportions; Wilson intervals quantify their endpoint sampling uncertainty. A competing-risk regression model was not fitted, because only 7 graft-loss events were available.

Analysis was limited to the first year because individual last-follow-up dates were unavailable. Computations used Python 3.12.14, pandas 2.2.3, NumPy 2.3.5, and SciPy 1.17.0, utilizing explicit likelihood and cumulative-incidence calculations.

### 2.6. Generative Artificial Intelligence Use

During manuscript revision, ChatGPT (model: GPT-6.1 Sol; OpenAI, San Francisco, CA, USA; https://chatgpt.com) was used to assist with language editing and formatting, generation of Python code for the statistical analyses described in Section 2.6, and revision of tables and statistical graphs to improve their clarity and consistency. The code-generation assistance related to the computations reported in Section 3 (Results); assistance with tables and graphs concerned the presentation of these analytical outputs. The study data were obtained from institutional clinical records, as described in Section 2.4.

The authors reviewed the generated code, edited it as needed, verified the analytical results, and reviewed the revised tables and graphs, their interpretations, and the final manuscript. Generative AI can produce incorrect code, unsupported statements, or biased suggestions; author review was therefore required before any output was incorporated. This assistance does not eliminate the possibility of coding errors, model misspecification, or bias in retrospective analyses. The authors retain full responsibility for the analytical decisions and the accuracy and integrity of the manuscript.

## 3. Results

### 3.1. Cohort and Data Availability

The cohort included 249 living-donor kidney transplant recipients. Mean recipient age was 36.4 ± 12.2 years, 153 recipients (61.4%) were male, mean donor age was 44.0 ± 11.2 years, and 88 donors (35.3%) were male. The cohort flow is shown in Figure 1.

Donor–recipient sex-pair counts are provided for both the full cohort and the primary analysis in Supplementary Table S6.

Mean recipient and donor BMI were 23.3 ± 4.4 and 27.2 ± 4.2 kg/m^2^, respectively. Mean recipient minus donor BMI difference was −3.9 ± 5.6 kg/m^2^, with a range from −19.31 to 14.06 kg/m^2^. Recipient BMI exceeded donor BMI in 63 pairs (25.3%). The median difference was −4.46 kg/m^2^ (IQR −7.32 to 0.03). Recipient BMI was <18.5 kg/m^2^ in 35 patients (14.1%) and ≥30 kg/m^2^ in 21 (8.4%); no donor had a BMI < 18.5 kg/m^2^, whereas 60 donors (24.1%) had a BMI ≥ 30 kg/m^2^. Complete BMI category distributions are shown in Table 1, and recipient–donor distributions are shown in Figure 2.

Preemptive transplantation was performed in 95 recipients (38.2%), 152 (61.0%) had received hemodialysis, and two (0.8%) had received peritoneal dialysis. Among previously dialyzed recipients, median dialysis duration was 10.5 months (IQR 2.0–37.5). Hypertension and diabetes mellitus were present in 110 (44.2%) and 10 (4.0%) recipients, respectively (Table 1).

Donor creatinine and derived eGFR were available for all 249 donors. Mean donor serum creatinine was 0.69 ± 0.11 mg/dL (range 0.39–1.05), and mean CKD-EPI 2021 eGFR was 111.2 ± 10.8 mL/min/1.73 m^2^ (range 71.3–139.5). All donors had no known kidney disease. All 249 recipients were PRA-negative and DSA-negative before transplantation, and every transplant was ABO-compatible. No recipient developed delayed graft function (0/249). Variable availability and qualitative clinical characteristics are summarized in Supplementary Table S1.

Rejection was recorded in 13 recipients (5.2%) during the first year: 12 had cellular rejection (4.8%) and one had mixed humoral and cellular rejection (0.4%). The median recorded time to rejection was 2.0 months (IQR 1.0–5.0; range 1–9). Of these recipients, 12 were included in the 1-year eGFR analysis and one died during the first year (Supplementary Table S7).

Valid eGFR measurements were available for 249 recipients at discharge, 240 at 6 months, and 235 at 1 year. By 6 months, nine recipients had experienced death or graft loss and had no valid 6-month graft-function measurement. Five recipients with valid 6-month measurements subsequently experienced death or graft loss before 1 year. Thus, all 14 recipients with first-year clinical events were excluded from the 1-year eGFR analysis. Mean recalculated eGFR values were 79.9 ± 27.4, 82.0 ± 21.5, and 81.3 ± 20.9 mL/min/1.73 m^2^ at discharge, 6 months, and 1 year, respectively. Clinical event analyses included all 249 recipients. No additional eGFR measurements were missing among recipients alive with a functioning graft at these visits. The primary analysis excluded no additional recipients for missing baseline covariates. Figure 1 shows the cohort flow; Supplementary Table S1 details measurement availability and fields not captured.

### 3.2. Primary eGFR Analysis

Recipient minus donor BMI difference was not statistically associated with 1-year eGFR in univariable analysis (B = −0.39, 95% CI −0.87 to 0.09; *p* = 0.111). After adjustment for the measured baseline covariates, higher BMI difference was associated with lower eGFR (B = −0.55 mL/min/1.73 m^2^ per 1 kg/m^2^, 95% CI −1.06 to −0.05; *p* = 0.033). On a 5 kg/m^2^ scale, the adjusted estimate was −2.77 mL/min/1.73 m^2^ (95% CI −5.31 to −0.23) (Table 3).

This estimate is conditional on survival with a functioning graft. Figure 3 shows observed and adjusted mean relationships, along with their uncertainty.

Complete covariate coefficients are reported in Supplementary Table S8; associations for variables other than the primary exposure are exploratory. Adjusted R^2^ was 0.195, the maximum variance inflation factor was 1.39, and the Breusch–Pagan *p* value was 0.897. These diagnostics do not exclude residual confounding or model instability.

### 3.3. Exploratory and Sensitivity Analyses

In the exploratory separate-BMI model, recipient and donor BMI coefficients were −0.55 (95% CI −1.24 to 0.14; *p* = 0.119) and 0.56 (95% CI −0.06 to 1.17; *p* = 0.078). Relaxing the equal-and-opposite restriction did not materially improve fit (F[1, 223] = 0.00011; *p* = 0.992). Adjusted R^2^ was 0.1948 versus 0.1912, and leave-one-out cross-validated RMSE was 19.28 versus 19.35 mL/min/1.73 m^2^ for the difference and separate-BMI models, respectively. Full fit comparisons are in Supplementary Table S2; additional functional-form and outcome specifications are in Supplementary Table S3.

Additional donor-eGFR adjustment gave a similar 1-year BMI-difference estimate (B = −0.55, 95% CI −1.06 to −0.05; *p* = 0.033; Supplementary Table S3). The adjusted 6-month estimate was −0.74 (95% CI −1.27 to −0.22; *p* = 0.006) in all 240 evaluable recipients and −0.68 (95% CI −1.20 to −0.15; *p* = 0.012) in the 235 recipients included at 1 year. These post hoc estimates concern different analysis populations and are compared descriptively (Table 2).

The post hoc mixed model used 724 measurements from 249 recipients (BMI-by-visit interaction: Wald χ^2^ = 11.41, 2 degrees of freedom; *p* = 0.003). Adjusted slopes per 1 kg/m^2^ BMI difference were −1.49 at discharge (95% CI −2.14 to −0.84; *p* < 0.001), −0.66 at 6 months (95% CI −1.16 to −0.17; *p* = 0.009), and −0.60 at 12 months (95% CI −1.10 to −0.10; *p* = 0.019). The discharge estimate requires particular caution because individual sampling dates were unavailable and a creatinine steady state could not be established. With common residual variance, the 12-month estimate was −0.53 (95% CI −1.08 to 0.03). Thus, its interval depended on the variance specification. Table 2 and Supplementary Table S4 report the estimates and 95% CIs; all longitudinal analyses were exploratory and unadjusted for multiple comparisons.

The ranked composite included nine recipients who died, five who were alive with graft loss, and 235 who were alive with a functioning graft. The unadjusted estimate was −1.86 percentile-rank points per 5 kg/m^2^ BMI difference (95% bootstrap CI −5.12 to 1.51); the adjusted estimate was −2.92 (95% bootstrap CI −6.39 to 0.41). Negative coefficients indicate a less favorable composite ranking with higher BMI difference, but both confidence intervals included zero (Supplementary Table S5).

### 3.4. Exploratory Clinical Outcomes

Within the first year, seven recipients experienced graft loss and nine died, with overlap in two recipients yielding 14 composite events among 249 recipients (5.6%). Estimated 12-month event-free survival was 94.4% (95% CI 90.7–96.6%). The exploratory unadjusted Cox hazard ratio per 5 kg/m^2^ BMI difference was 0.88 (95% CI 0.55–1.42; *p* = 0.608). The time-interaction test did not detect nonproportionality (*p* = 0.677), although only 14 events were available (Table 3).

In the competing-risk analysis, seven recipients had graft loss as their first event and seven died before graft loss. The 12-month cumulative incidence was 2.81% for graft loss (95% CI 1.37–5.69%) and 2.81% for death before graft loss (95% CI 1.37–5.69%). The two deaths following graft loss were not counted again as competing first events (Figure 4).

Of the nine first-year deaths, six were attributed to infection, two to myocardial infarction, and one to a cerebrovascular event. One infection-related death and one myocardial-infarction death followed graft loss. Recorded causes and denominators are detailed in Supplementary Table S9.

## 4. Discussion

Higher recipient minus donor BMI difference was associated with lower 1-year eGFR after adjustment for measured covariates. A 5 kg/m^2^ higher difference corresponded to −2.77 mL/min/1.73 m^2^ (95% CI −5.31 to −0.23). Although the adjusted interval excluded zero, it spans small to potentially appreciable decrements; no clinically meaningful margin was prespecified. Statistical significance therefore does not establish clinical usefulness or a donor-selection threshold. The unadjusted and laboratory-reported-eGFR estimates had intervals including zero, and the ranked-composite estimate was imprecise. The primary association applies specifically to survivors with functioning grafts.

Nephron underdosing concerns insufficient nephron supply relative to recipient requirements and possible adaptive hyperfiltration [7,8,9]. Previous studies have examined donor–recipient weight/BSA ratios, kidney size, and sex mismatch, with associations varying by metric, donor type, and outcome [10,11,12]. Those findings provide context, but this study measured neither nephron mass nor hyperfiltration and cannot test the mechanism directly. Its exposure is an arithmetic BMI difference, not a validated measure of anatomical donor–recipient size mismatch.

BMI cannot distinguish lean mass from adiposity or quantify kidney volume, kidney weight, nephron number, or kidney-specific functional reserve. Identical BMIs can occur at different heights and absolute body sizes; CT-derived kidney volume provides anatomical information that BMI cannot reproduce [13]. Source height, weight, and renal anatomical measurements were unavailable, precluding weight/BSA ratios and anatomical validation. Only approximately one-quarter of pairs had a positive BMI difference, and sparse extreme positive values were recorded. This sign does not establish kidney undersizing, and effects in recipients with marked positive differences remain uncertain.

Recipient and donor obesity may affect outcomes through different pathways [14,15,16,17,18,19]. Similar fit and internal prediction error for the difference and separate-BMI models do not establish biological equivalence or superiority of subtraction. Neither component coefficient individually met the nominal significance threshold, but this does not contradict the greater precision of the one-parameter constrained estimate. Failure to reject the equal-and-opposite restriction likewise does not validate it biologically.

Secondary covariate findings, including donor age [6,26] and recipient age or sex, should be interpreted cautiously. The unexpected preemptive-status coefficient is a model contrast at zero dialysis duration, rather than an average between-group difference. Residual confounding, selection, or model specification may explain it. This exploratory result should not be interpreted causally or as evidence against the established benefits of preemptive transplantation [27].

Residual confounding remains plausible despite adjustment. Donor serum creatinine and derived eGFR were available, but measured GFR, 24 h creatinine clearance, and split renal function were unavailable in the original clinical records. Adding donor eGFR did not materially alter the BMI estimate, but this metric cannot fully characterize the functional reserve of the transplanted kidney. Cold/warm ischemia times, recipient primary kidney disease, detailed comorbidity severity, and individual immunosuppressive exposure were unavailable. Hypertension and diabetes indicators do not capture severity or control. These limitations prevent a causal interpretation of the adjusted coefficient.

Only 14 composite events and seven graft losses occurred, precluding reliable adjusted Cox or competing-risk regression. The exploratory unadjusted HR of 0.88 (95% CI 0.55–1.42) remains compatible with appreciable associations in either direction and cannot guide donor selection. Month-based event timing limits temporal precision, and unavailable last-follow-up dates prevented longer-term analyses.

All secondary and sensitivity analyses were exploratory and unadjusted for multiple comparisons. Nominal visit dates could not be verified, and discharge creatinine may not have been at steady state. Cystatin C was not available and was not incorporated into eGFR calculation. Reliance on creatinine-based eGFR may introduce non-GFR influences related to muscle mass and body composition, which are particularly relevant when studying BMI. Combined creatinine–cystatin C estimates have been evaluated against measured GFR in transplant recipients [28], but corticosteroids, inflammation, and adiposity can also affect cystatin C [29]. Larger studies should document anatomical measurements, body composition, and repeated multimarker graft function.

## 5. Conclusions

Higher recipient minus donor BMI difference was associated with lower 1-year creatinine-based eGFR after adjustment for measured covariates in the 235 recipients alive with functioning grafts. Complementary full-cohort analyses concern different outcomes and do not change the conditional interpretation of this eGFR finding. All secondary analyses were exploratory and unadjusted for multiple comparisons. Residual confounding and sparse clinical events prevent causal or graft-survival conclusions. BMI difference is an anthropometric exposure; these findings do not validate anatomical size mismatch or justify donor-selection thresholds.

## Figures and Tables

**Figure 1:**
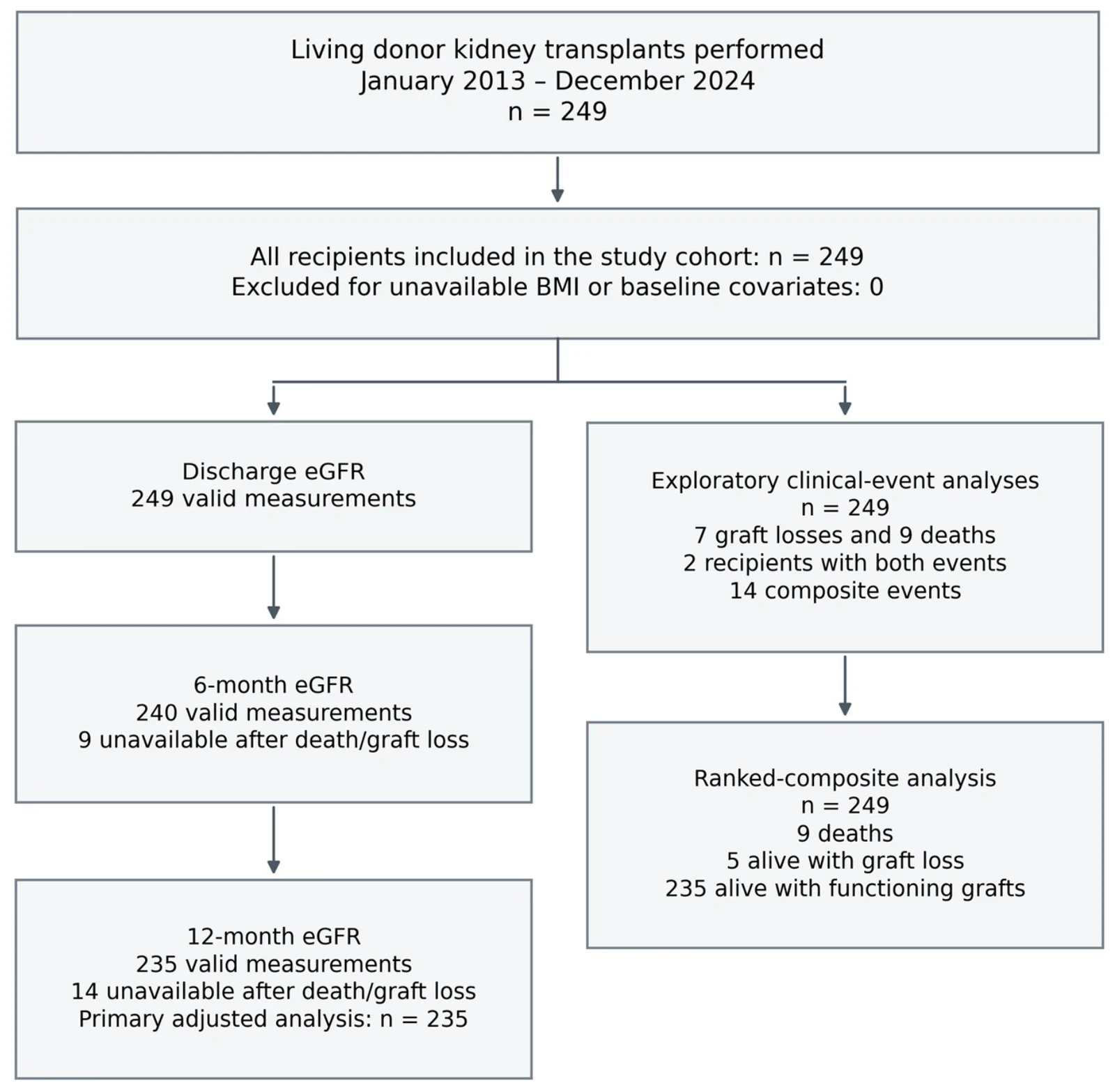
Cohort inclusion and analysis populations. All 249 living-donor kidney transplant recipients at the center during January 2013December 2024 were included. No recipient was excluded because BMI or required baseline covariate data were unavailable. Valid eGFR measurements were available for 249 recipients at discharge, 240 at 6 months, and 235 at 12 months. Death or graft loss accounted for all nine unavailable 6month measurements and all 14 unavailable 12-month measurements. The primary adjusted eGFR analysis included 235 recipients alive with functioning grafts; clinical-event and ranked-composite analyses retained all 249. The ranked composite comprised nine deaths, five recipients alive with graft loss, and 235 alive with functioning grafts. BMI, body mass index; eGFR, estimated glomerular filtration rate.

**Figure 2:**
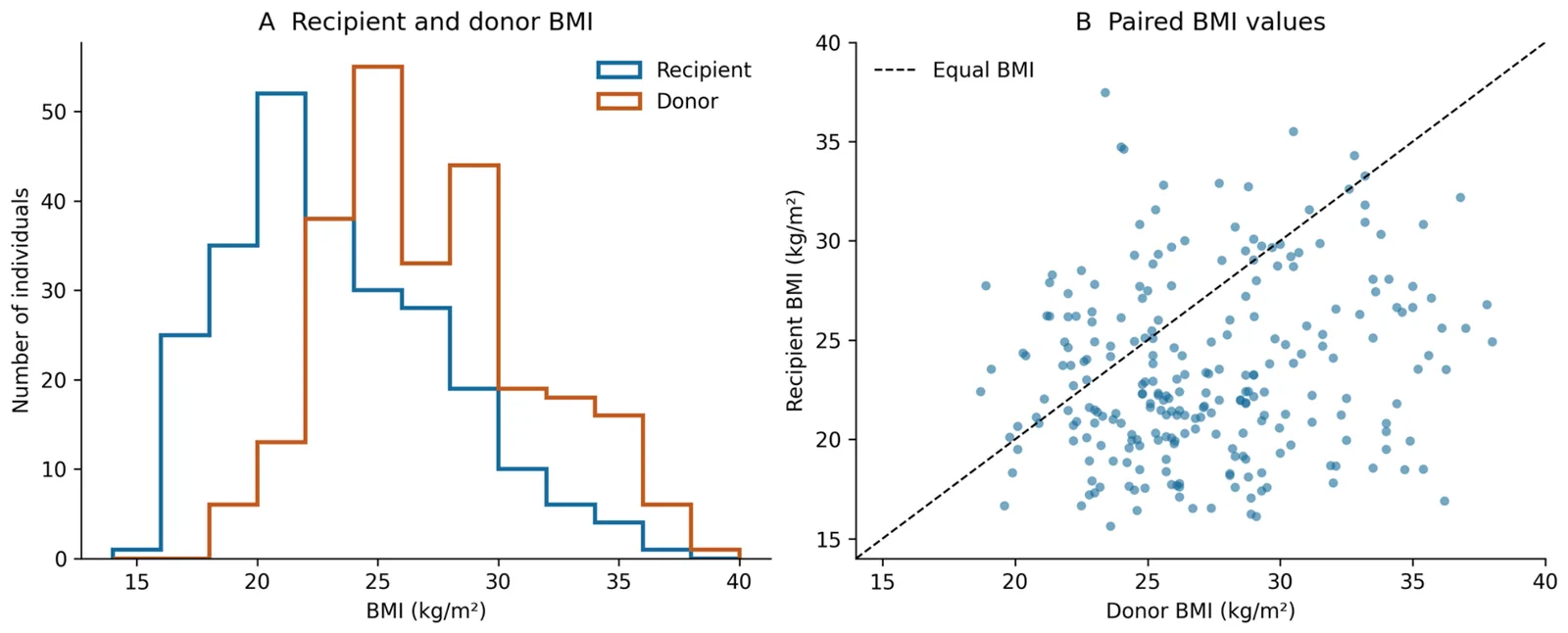
Recipient and donor BMI distributions in 249 pairs. (**A**) shows histograms with identical bin widths. (**B**) shows paired values; the dashed diagonal denotes equal BMI. The plot describes anthropometric distributions and provides no measurement of kidney or cortical volume. BMI, body mass index.

**Figure 3:**
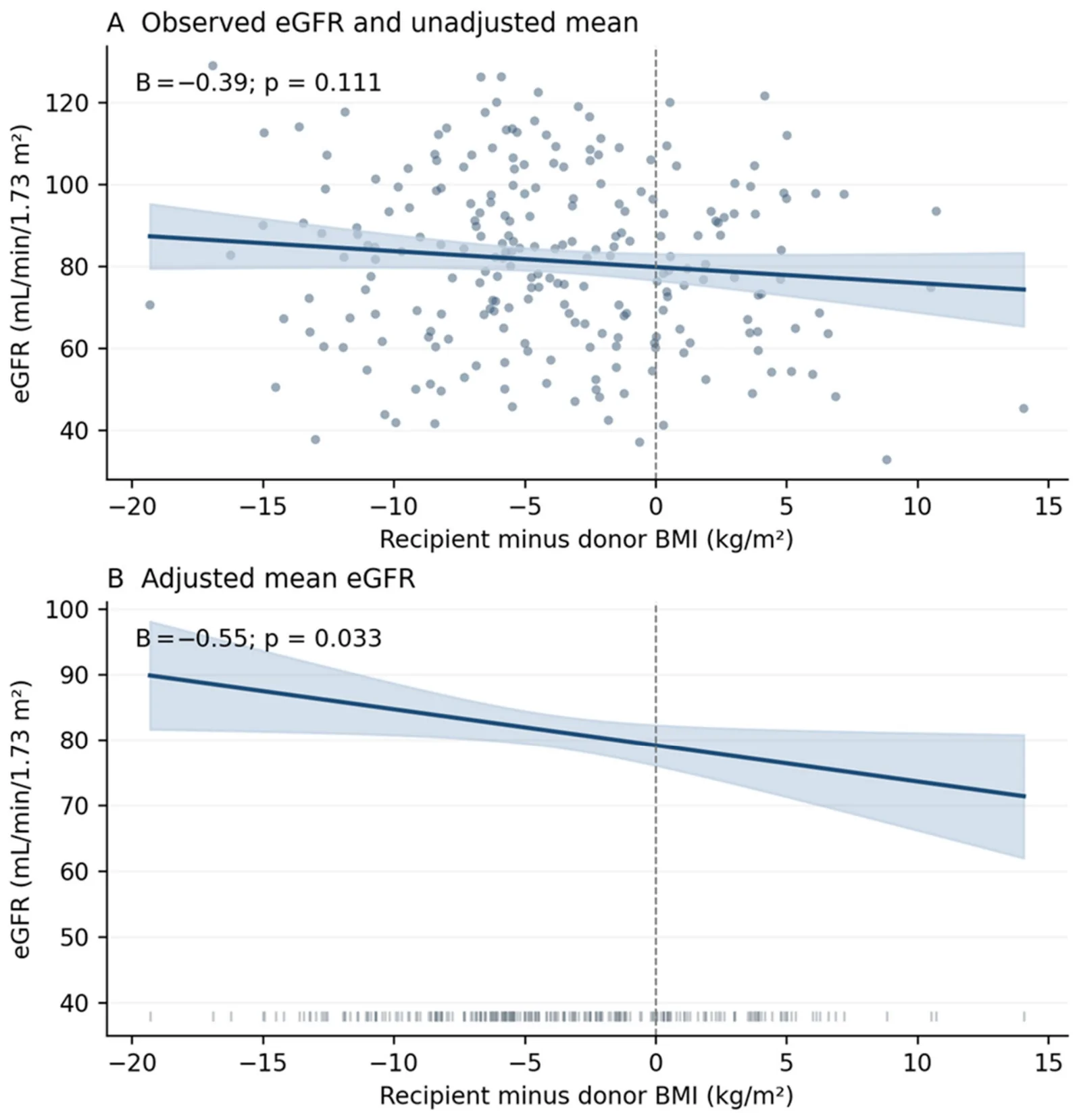
Recipient minus donor BMI difference and 1-year graft function in 235 recipients alive with a functioning graft. (**A**) Dots show individual 2021 CKD-EPI eGFR values, and the solid blue line shows the unadjusted linear fitted mean. (**B**) The solid blue line shows adjusted mean predictions averaged over the cohort’s observed baseline covariates: recipient and donor age and sex, dialysis duration, preemptive status, HLA mismatch, hypertension, and diabetes. Shaded bands show pointwise 95% confidence intervals for the mean, not individual prediction intervals. Rug marks in (**B**) show observed BMI differences; the vertical dashed line at zero in both panels indicates equal recipient and donor BMI. B denotes the regression coefficient, expressed as the change in eGFR (mL/min/1.73 m^2^) per 1 kg/m^2^ increase in recipient minus donor BMI difference; p denotes the two-sided p value for the corresponding regression coefficient. No eGFR was assigned after death or graft loss. BMI, body mass index; eGFR, estimated glomerular filtration rate; CKD-EPI, Chronic Kidney Disease Epidemiology Collaboration; HLA, human leukocyte antigen.

**Figure 4:**
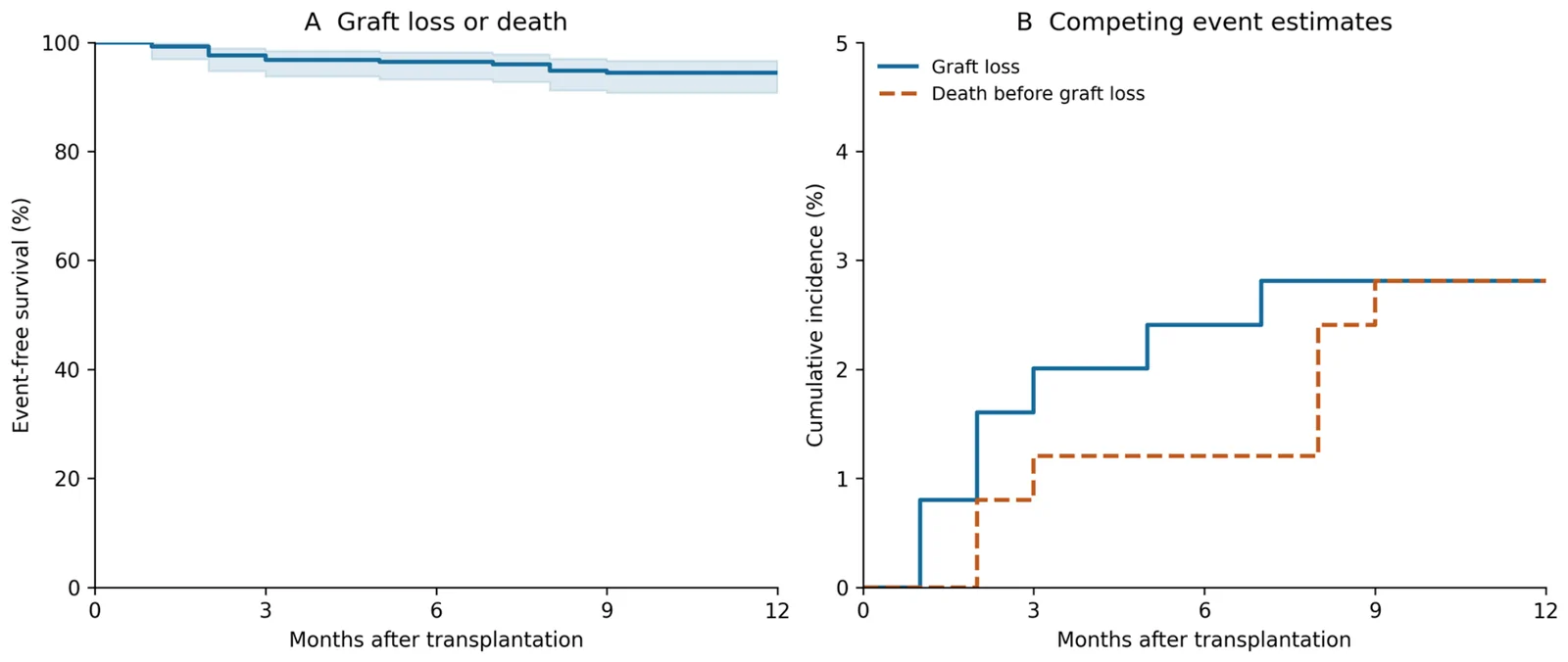
First-year outcomes among all 249 recipients. (**A**) shows Kaplan–Meier survival free of graft loss or death; shading denotes its 95% confidence interval. (**B**) shows Aalen–Johansen cumulative incidences for graft loss and death before graft loss. The two recipients who experienced graft loss followed by death contributed graft-loss events. Numbers at risk immediately before months 0, 3, 6, 9, and 12 were 249, 243, 240, 236, and 235.

**Table 1:** Baseline characteristics and 1-year outcomes.

Variable	Overall (n = 249)
Recipient age, years	36.4 ± 12.2
Male recipient, n (%)	153 (61.4)
Donor age, years	44.0 ± 11.2
Donor serum creatinine, mg/dL	0.69 ± 0.11
Donor CKD-EPI 2021 eGFR, mL/min/1.73 m^2^	111.2 ± 10.8
Male donor, n (%)	88 (35.3)
Recipient BMI, kg/m^2^	23.3 ± 4.4
Donor BMI, kg/m^2^	27.2 ± 4.2
Recipient BMI < 18.5 kg/m^2^, n (%)	35 (14.1)
Recipient BMI 18.5–<25 kg/m^2^, n (%)	135 (54.2)
Recipient BMI 25–<30 kg/m^2^, n (%)	58 (23.3)
Recipient BMI ≥ 30 kg/m^2^, n (%)	21 (8.4)
Donor BMI < 18.5 kg/m^2^, n (%)	0 (0.0)
Donor BMI 18.5–<25 kg/m^2^, n (%)	82 (32.9)
Donor BMI 25–<30 kg/m^2^, n (%)	107 (43.0)
Donor BMI ≥ 30 kg/m^2^, n (%)	60 (24.1)
Recipient minus donor BMI difference, kg/m^2^	−3.9 ± 5.6
BMI-difference range, kg/m^2^	−19.31 to 14.06
Recipient BMI > donor BMI, n (%)	63 (25.3)
Preemptive transplantation, n (%)	95 (38.2)
Hemodialysis, n (%)	152 (61.0)
Peritoneal dialysis, n (%)	2 (0.8)
Dialysis duration among dialyzed recipients, months	10.5 (2.0–37.5)
HLA mismatch number	3.2 ± 1.9
Recipient hypertension, n (%)	110 (44.2)
Recipient diabetes mellitus, n (%)	10 (4.0)
Valid 1-year CKD-EPI eGFR, mL/min/1.73 m^2^	81.3 ± 20.9 (n = 235)
Graft loss within 1 year, n (%)	7 (2.8)
Death within 1 year, n (%)	9 (3.6)
Graft loss or death within 1 year, n (%)	14 (5.6)

Values are mean ± SD, median (IQR), or n (%). Baseline and clinical-event denominator: 249. The primary eGFR denominator was 235. Two recipients experienced graft loss followed by death and are included in both component counts but only once in the composite. BMI, body mass index; eGFR, estimated glomerular filtration rate; HLA, human leukocyte antigen. Preemptive recipients had a dialysis duration of 0 months; the duration summary refers only to previously dialyzed recipients. Variable-level availability is reported in Supplementary Table S1. SD, standard deviation; IQR, interquartile range; CKD-EPI, Chronic Kidney Disease Epidemiology Collaboration.

**Table 2:** Exploratory 6-month and longitudinal eGFR analyses.

Analysis	B	95% CI	*p* Value
6-month eGFR, all available (n = 240)	−0.74	−1.27 to −0.22	0.006
6-month eGFR, 1-year analysis population (n = 235)	−0.68	−1.20 to −0.15	0.012
Mixed model: discharge	−1.49	−2.14 to −0.84	<0.001
Mixed model: 6 months	−0.66	−1.16 to −0.17	0.009
Mixed model: 12 months	−0.60	−1.10 to −0.10	0.019
Mixed model: BMI-by-visit interaction	—	—	0.003

B is eGFR difference (mL/min/1.73 m^2^) per 1 kg/m^2^ BMI difference. All analyses were post hoc, used the original baseline covariates, and were unadjusted for multiple comparisons. The random-intercept mixed model included 724 measurements from 249 recipients and allowed visit-specific residual variances. Its slope CIs are Wald intervals; the interaction *p* value is a joint 2-degree-of-freedom Wald test. Discharge results require caution because sampling dates and creatinine steady-state status were unavailable. No post-event eGFR was imputed. Both variance specifications are detailed in Supplementary Table S4; additional 1-year sensitivity analyses are in Supplementary Table S3. BMI, body mass index; eGFR, estimated glomerular filtration rate; CI, confidence interval.

**Table 3:** Primary eGFR association and exploratory first-year clinical outcomes.

Outcome or Model	Estimate	95% CI	*p* Value
A. Primary 1-year eGFR analysis (n = 235)			
Unadjusted, per 1 kg/m^2^	B −0.39	−0.87 to 0.09	0.111
Adjusted, per 1 kg/m^2^	B −0.55	−1.06 to −0.05	0.033
Adjusted, per 5 kg/m^2^	B −2.77	−5.31 to −0.23	0.033
B. Exploratory first-year clinical outcomes (n = 249; 14 events)			
Cox model, per 5 kg/m^2^ BMI difference	HR 0.88	0.55–1.42	0.608
12-month event-free survival	94.4%	90.7–96.6%	—
12-month graft-loss cumulative incidence	2.81%	1.37–5.69%	—
12-month cumulative incidence of death before graft loss	2.81%	1.37–5.69%	—

Panel A: B is the eGFR difference (mL/min/1.73 m^2^) per indicated BMI-difference increment. Adjustment included recipient/donor age and sex, dialysis duration, preemptive status, HLA mismatch, hypertension, and diabetes. The per-5 kg/m^2^ estimate and CI were rescaled from unrounded per-unit estimates; they describe the same fitted model and *p* value. Panel B: all clinical-event estimates are exploratory. HR is the unadjusted Cox hazard ratio (Efron ties). Kaplan–Meier event-free survival used log–log CIs; Aalen–Johansen cumulative incidences used Wilson endpoint intervals because there was no censoring before 12 months. Two deaths after graft loss contributed graft-loss first events. BMI, body mass index; eGFR, estimated glomerular filtration rate; CI, confidence interval; HLA, human leukocyte antigen.

## Data Availability

The de-identified data that support the findings of this study are available from the corresponding author upon reasonable request, subject to institutional and ethical approval.

## Supplementary Materials

The following supporting information can be downloaded at: https://www.mdpi.com/article/10.3390/jcm15218298/s1. Table S1: Variable-level data availability; Table S2: Comparison of BMI parameterizations; Table S3: Additional 1-year eGFR sensitivity analyses; Table S4: Longitudinal coefficients and confidence intervals; Table S5: Full-cohort ranked composite; Table S6: Donor–recipient sex pairs; Table S7: Post-transplant rejection; Table S8: Full primary multivariable eGFR model; Table S9: Causes of first-year death.
