# Supplementary material for: Recipient Minus Donor Body Mass Index Difference and One-Year Graft Function After Living-Donor Kidney Transplantation
Source: J Clin Med. 2026 Oct 25;15(21):8298. doi: 10.3390/jcm15218298 (PMC13764344; doi:10.3390/jcm15218298)
Supplement: Supplementary file 1 [file jcm-15-08298-s001.zip › jcm-4632280-supplementary.pdf]

Supplementary material

Recipient Minus Donor Body Mass Index Difference and One Year Graft Function After Living Donor Kidney Transplantation

Supplementary Table S1. Variable-level data availability

A. Baseline variables

Variable	Available / 249	Missing / 249	Missing / 235[1]
Recipient age	249	0	0
Recipient sex	249	0	0
Donor age	249	0	0
Donor serum creatinine	249	0	0
Donor eGFR derived from creatinine	249	0	0
Donor sex	249	0	0
Recipient BMI	249	0	0
Donor BMI	249	0	0
Recipient-minus-donor BMI difference	249	0	0
Dialysis duration	249	0	0
Preemptive transplantation	249	0	0
HLA mismatch number	249	0	0
Recipient hypertension	249	0	0
Recipient diabetes mellitus	249	0	0
Pretransplant dialysis modality	249	0	0

[1] All 249 living donor transplant recipients were included; none were excluded for unavailable BMI or required baseline covariates. The 235 recipients with valid 12-month eGFR formed the primary multivariable analysis population and had complete included baseline variables. Complete recording does not address unmeasured confounding.

Dialysis duration was 0 months in all 95 preemptive recipients. The remaining 154 had received hemodialysis (152) or peritoneal dialysis (2). Joint inclusion of duration and preemptive status permits a duration slope among dialyzed recipients and a status contrast at zero months, not the average group difference.

BMI, body mass index; eGFR, estimated glomerular filtration rate; HLA, human leukocyte antigen.

Supplementary Table S1 (continued)

B. Graft function, event status, and event timing

Variable	Available / 249	Unavailable / 249	Missing / 235[1]
Discharge recipient creatinine	249	0	0
Discharge laboratory-reported recipient eGFR	249	0	0
Discharge recalculated recipient eGFR	249	0	0
6 months recipient creatinine	240	9	0
6 months laboratory-reported recipient eGFR	240	9	0
6 months recalculated recipient eGFR	240	9	0
12 months recipient creatinine	235	14	0
12 months laboratory-reported recipient eGFR	235	14	0
12 months recalculated recipient eGFR	235	14	0
Graft-loss status	249	0	0
Death status	249	0	0
Post-transplant rejection status	249	0	0

At 6 months, 9 measurements were unavailable after death or graft loss; at 12 months, 14 were unavailable for this reason. No additional eGFR measurements were missing among recipients alive with a functioning graft. These are outcomes truncated by clinical events, not sporadically missing baseline values. No post-event eGFR was imputed.

Conditional field	Applicable	Observed	Missing if applicable	Not applicable
Month of graft loss	7	7	0	242
Month of death	9	9	0	240
Recorded rejection type	13	13	0	236
Month of recorded rejection	13	13	0	236
Recorded cause of first-year death	9	9	0	240

Event-specific fields apply only to recipients with that event. Blank rejection entries indicate no rejection; type and month were not applicable in 236 recipients. All applicable rejection types, months, and first-year death causes were complete. Mortality summaries cover the first post-transplant year and do not characterize later outcomes. There were 9 deaths, 7 graft losses, and 14 recipients with either event; two had graft loss followed by death. Blank event-month cells in recipients without a recorded event are not missing applicable times.

Supplementary Table S1 (continued)

C. Qualitative clinical characteristics and unavailable variables

Qualitative clinical characteristic	n / 249
Donor creatinine within the normal range	249 / 249
Known donor kidney disease	0 / 249
PRA-positive recipients	0 / 249
DSA-positive recipients	0 / 249
ABO-compatible transplantation	249 / 249
Delayed graft function	0 / 249

All 249 transplants were first living related donor kidney transplants; no marginal donors were included. These cohort characteristics were assessed qualitatively; PRA negativity was not assigned a numerical cPRA value.

Donor and recipient BMI were assessed immediately before transplantation, using the most recent dry weight in dialysis recipients. Individual measurement dates were not retained.

Remaining unavailable field	Individual numerical / clinical records
Recipient primary kidney disease	Not available
Quantitative PRA/cPRA results and antibody assay details	Not available
Patient-level induction receipt, duration and cumulative dose	Not available
Longitudinal maintenance doses and measured drug levels	Not available
Rejection biopsy confirmation and histological grading	Not available
Major immunosuppressive treatment changes	Not available
Cold ischemia time	Not available
Warm ischemia time	Not available
Measured donor GFR, creatinine clearance, or split renal function	Unavailable in original clinical records

Induction selection, protocol doses, tacrolimus targets and steroid tapering are described in the main Methods. Patient-level induction exposure, longitudinal doses/levels, alternative agents (including everolimus) and treatment changes were unavailable. Rejection therapy was type-directed; type and month were recorded, but biopsy grading and episode-specific treatments were unavailable (Supplementary Table S7). Grafts were transplanted promptly after living donor nephrectomy; individual ischemia times were unavailable. Protocol targets do not establish achieved exposure.

Source height and weight, waist circumference, body composition, kidney/cortical volume, kidney weight, cystatin C, and measured GFR were unavailable in the analytic dataset. Weight/BSA comparisons and anatomical validation were therefore not possible. Donor serum creatinine and derived CKD-EPI 2021 eGFR were available for all 249 donors. No other donor functional

measurements were available in the original clinical records. Recorded death causes are in Supplementary Table S9; detailed infection diagnoses and cardiovascular subtypes were unavailable.

PRA, panel-reactive antibodies; cPRA, calculated PRA; DSA, donor-specific antibodies; ABO, blood-group compatibility.

Supplementary Table S2. Comparison of BMI parameterizations

Both models used the same 235 recipients, 1-year CKD-EPI 2021 eGFR outcome, and original baseline covariates. The difference model imposes equal-magnitude, opposite-sign recipient and donor BMI coefficients. The separate-BMI model estimates both coefficients freely. Lower AIC, BIC, and RMSE indicate better fit or lower prediction error on the specified scale. The original primary specification was not selected using these comparisons.

Metric	BMI difference	Separate recipient and donor BMI
Recipients	235	235
Regression coefficients including intercept	11	12
R^2	0.229237	0.229237
Adjusted R^2	0.1948	0.1912
AIC	2058.46	2060.46
BIC	2099.97	2105.43
In-sample RMSE, mL/min/1.73 m^2	18.351	18.351
Leave-one-out cross-validated RMSE, mL/min/1.73 m^2	19.277	19.354

The equal-and-opposite restriction test gave $F(1, 223) = 0.00011$, $p = 0.992$. In the unrestricted model, the sum of the recipient and donor BMI coefficients was 0.004 (95% CI −0.814 to 0.823). AIC and BIC count the residual-variance parameter in addition to regression coefficients. Leave-one-out RMSE was calculated from each omitted observation's prediction error using the OLS leverage identity. This is internal cross-validation, not external validation. Failure to reject the restriction does not establish biological equality of effects.

Supplementary Table S3. Additional 1-year eGFR sensitivity analyses

All analyses were post hoc and exploratory and used the same 235 recipients with valid 1-year graft-function measurements and the original baseline covariates. The donor-eGFR model additionally included donor eGFR. Recipient and donor BMI were entered together in the separate-BMI model.

**Analysis or exposure**	**B**	**95% CI**	**p value**
Recipient BMI, with donor BMI in the same model	−0.55	−1.24 to 0.14	0.119
Donor BMI, with recipient BMI in the same model	0.56	−0.06 to 1.17	0.078

Analysis or exposure	B	95% CI	p value
Positive vs nonpositive BMI difference	−5.67	−11.70 to 0.35	0.065
Quadratic and cubic terms: joint nonlinearity test	—	—	0.653
BMI difference with additional donor-eGFR adjustment	−0.55	−1.06 to −0.05	0.033
Laboratory-reported eGFR: continuous BMI difference	−0.44	−0.93 to 0.04	0.073

B is eGFR difference in mL/min/1.73 m² per 1 kg/m² unless a binary comparison is specified. The joint nonlinearity test assesses the added quadratic and cubic terms; it has no single coefficient or CI. Nominal p values were not adjusted for multiple comparisons. Full model-fit comparisons are in Supplementary Table S2, and both longitudinal variance specifications are in Supplementary Table S4. BMI, body mass index; eGFR, estimated glomerular filtration rate; CI, confidence interval.

Supplementary Table S4. Longitudinal coefficients and confidence intervals

These post hoc random-intercept models used 724 pre-event measurements from all 249 recipients, categorical visit, BMI-by-visit interactions, and the original baseline adjustment covariates. Variance components were estimated by restricted maximum likelihood. B is eGFR difference in mL/min/1.73 m² per 1 kg/m² higher BMI difference. Coefficient CIs are two-sided 95% Wald intervals.

Residual variance	Visit	B	95% CI	p value
Visit-specific	Discharge	−1.49	−2.14 to −0.84	<0.001
Visit-specific	6 months	−0.66	−1.16 to −0.17	0.009
Visit-specific	12 months	−0.60	−1.10 to −0.10	0.019
Common	Discharge	−1.46	−2.00 to −0.91	<0.001
Common	6 months	−0.60	−1.15 to −0.05	0.032
Common	12 months	−0.53	−1.08 to 0.03	0.062

BMI-by-visit interaction: visit-specific residual variances, Wald $\chi^2(2) = 11.41$, $p = 0.003$; common residual variance, Wald $\chi^2(2) = 18.40$, $p < 0.001$. Confidence intervals do not apply to these omnibus tests. All analyses were exploratory and unadjusted for multiple comparisons. The likelihood assumes an ignorable observation process conditional on the model; neither variance specification eliminates informative truncation by death or graft loss. Individual measurement dates were unavailable, and discharge creatinine steady state could not be established.

Supplementary Table S5. Full-cohort ranked composite

All 249 recipients contributed to this post hoc exploratory analysis. Higher scores represent a more favorable 12-month clinical outcome. The outcome hierarchy was fixed for this sensitivity analysis before fitting the models.

Outcome category at 12 months	n	Ranking rule
Death	9	Tied worst rank

Outcome category at 12 months	n	Ranking rule
Alive with graft loss	5	Tied above death, below functioning graft
Alive with a functioning graft	235	Ordered by increasing 12-month eGFR

Both recipients with graft loss followed by death were classified in the death category. This differs from a first-event competing-risk analysis, in which each contributes a graft-loss event.

Model	n	B per 5 kg/m²	95% bootstrap CI
Unadjusted	249	−1.86	−5.12 to 1.51
Adjusted[1]	249	−2.92	−6.39 to 0.41

B is measured in percentile-rank points, not mL/min/1.73 m². The midrank was scaled as 100 × (rank − 0.5)/249. The continuous exposure remained recipient-minus-donor BMI difference. Percentile 95% confidence intervals used 5,000 nonparametric participant-level bootstrap resamples (seed 20260930), with ranks recalculated within each resample.

[1] Adjusted for recipient and donor age and sex, dialysis duration, preemptive transplantation, HLA mismatch number, hypertension, and diabetes mellitus. All exposure and adjustment variables were complete for all 249 recipients. BMI, body mass index; CI, confidence interval; eGFR, estimated glomerular filtration rate; HLA, human leukocyte antigen.

This analysis incorporates clinical events without assigning a post-event eGFR. It is a composite association conditional on the selected hierarchy; it does not estimate a causal effect or eliminate unmeasured confounding. Its confidence interval includes effects in either direction. Because 235 recipients had functioning grafts, the ranking is predominantly determined by eGFR among survivors.

Methodological reference: Lachin JM. Worst-rank score analysis with informatively missing observations in clinical trials. Control Clin Trials. 1999;20(5):408–422. doi:10.1016/S0197-2456(99)00022-7.

Supplementary Table S6. Donor–recipient sex pairs

Pair counts describe the full cohort and the 235 recipients included in the primary 1-year eGFR model. The direction is donor → recipient.

Sex pair	Full cohort<br>n = 249	Primary analysis<br>n = 235
Male donor → male recipient	38 (15.3%)	37 (15.7%)
Female donor → male recipient	115 (46.2%)	105 (44.7%)
Male donor → female recipient	50 (20.1%)	48 (20.4%)
Female donor → female recipient	46 (18.5%)	45 (19.1%)

Percentages use the corresponding column denominator. Totals are 249 and 235; minor rounding differences may occur. These are descriptive counts, not an interaction analysis.

Donor and recipient sex were included as main-effect covariates. This adjustment does not test whether the relationship between BMI difference and eGFR varies by sex mismatch. No sex-pair subgroup or interaction model was fitted.

Supplementary Table S7. Post-transplant rejection

Rejection during the first year after transplantation in the full cohort of 249 recipients. Counts refer to recipients with recorded rejection, not to the total number of episodes.

Characteristic	Result
Any recorded rejection, n (%)	13 (5.2)
Cellular rejection, n (%)	12 (4.8)
Mixed humoral and cellular rejection, n (%)	1 (0.4)
No rejection, n (%)	236 (94.8)
Recorded month of rejection, median (IQR)	2.0 (1.0–5.0)
Recorded month of rejection, range	1–9

All percentages use the full-cohort denominator of 249. Timing summaries use the 13 recipients with rejection and are expressed in months after transplantation. Blank rejection entries represented no rejection. All recorded rejection types and applicable months were complete. IQR, interquartile range.

Twelve recipients with rejection were included in the 235-person 1-year eGFR analysis. One recipient had cellular rejection recorded in month 1 and died in month 3; this recipient contributed to the full-cohort event and ranked-composite analyses. The sequence does not establish that rejection caused death.

Clinical type labels were retained as recorded. Biopsy confirmation, histological grades, repeated episodes, and episode-specific treatment details were not available. Rejection was reported descriptively and was not added to the primary model of baseline factors.

Supplementary Table S8. Full primary multivariable eGFR model

The original primary model used 235 recipients alive with functioning grafts at 12 months. The continuous BMI-difference term was the primary exposure; other covariate estimates are exploratory. All included variables were complete.

Variable	B	95% CI	p value
Recipient-minus-donor BMI difference, per kg/m^2	−0.55	−1.06 to −0.05	0.033
Recipient age, per year	−0.21	−0.44 to 0.02	0.072
Donor age, per year	−0.73	−0.95 to −0.50	<0.001
Male recipient vs female	−5.08	−10.36 to 0.20	0.059
Male donor vs female	5.93	0.56 to 11.31	0.031
Dialysis duration, per month	−0.02	−0.08 to 0.05	0.586
Preemptive vs dialyzed, at 0 months[1]	−6.13	−11.58 to −0.69	0.028

Variable	B	95% CI	p value
HLA mismatch, per mismatch	−0.94	−2.28 to 0.39	0.166
Hypertension, yes vs no	−0.38	−5.60 to 4.83	0.885
Diabetes mellitus, yes vs no	5.13	−7.40 to 17.66	0.421

B values are unstandardized eGFR differences in mL/min/1.73 m² per unit or comparison indicated. Adjusted $R^2 = 0.195$. Secondary covariate estimates and nominal p values do not establish causal effects. [1] With dialysis duration coded as zero in preemptive recipients, this coefficient is the fitted status contrast at zero months, not an average group difference. BMI, body mass index; eGFR, estimated glomerular filtration rate; CI, confidence interval; HLA, human leukocyte antigen.

Supplementary Table S9. Causes of first-year death

Recorded causes were available for every first-year death. The full cohort comprised 249 recipients; 9 died during the first year.

Recorded cause	n	Among 9 deaths (%)	Among 249 recipients (%)
Infection	6	66.7	2.4
Myocardial infarction	2	22.2	0.8
Cerebrovascular event	1	11.1	0.4

One infection-related death and one myocardial-infarction death occurred after graft loss; the remaining seven deaths occurred without prior recorded graft loss. The two deaths after graft loss belong to the death category in the ranked composite, but contribute graft-loss first events in the competing-risk analysis.

Causes of death are reported as recorded. More specific infectious diagnoses and cerebrovascular subtypes were unavailable. No cause was inferred from rejection or graft loss. The sequence of clinical events does not establish causation, and no cause-specific regression was fitted.
